# α-Pyrrolidinooctanophenone facilitates activation of human microglial cells via ROS/STAT3-dependent pathway

**DOI:** 10.1007/s11419-024-00708-x

**Published:** 2024-12-09

**Authors:** Yuji Sakai, Junta Hattori, Yoshifumi Morikawa, Toshihiro Matsumura, Shunsuke Jimbo, Koichi Suenami, Tomohiro Takayama, Atsushi Nagai, Tomomi Michiue, Akira Ikari, Toshiyuki Matsunaga

**Affiliations:** 1 Forensic Science Laboratory, Gifu Prefectural Police Headquarters, Gifu, 500-8501 Japan; 2 Laboratory of Bioinformatics, Gifu Pharmaceutical University, Gifu, 502-8585 Japan; 3 Department of Legal Medicine, Graduate School of Medicine, Gifu University, Gifu, 501-1194 Japan; 4 Laboratory of Biochemistry, Gifu Pharmaceutical University, Gifu, 501-1196 Japan

**Keywords:** α-Pyrrolidinooctanophenone, Methamphetamine, Microglial cell activation, Reactive oxygen species, Interleukin-6, Signal transducer and activator of transcription 3

## Abstract

**Purpose:**

Pyrrolidinophenone derivatives (PPs) are amphetamine-like designer drugs containing a pyrrolidine ring, and their adverse effects resemble those of methamphetamine (METH). Microglial activation has been recently suggested as a key event in eliciting the adverse effects against dysfunction of the central nervous system. The aim of this study is to clarify the mechanisms of microglial activation induced by PPs.

**Methods:**

We employed the human microglial cell line HMC3 to assess microglial activation induced by PPs and evaluated the capacities for proliferation and interleukin-6 (IL-6) production that are characteristic features of the activation events.

**Results:**

The WST-1 assay indicated that viability of HMC3 cells was increased by treatment with sublethal concentrations (5–20 µM) of α-pyrrolidinooctanophenone (α-POP), a highly lipophilic PP, whereas it was decreased by treatment with concentrations above 40 µM. Treatment with sublethal α-POP concentrations up-regulated the expression and secretion of IL-6. Additionally, α-POP-induced increase in cell viability was restored by pretreating with *N*-acetyl-l-cysteine, a reactive oxygen species (ROS) scavenger, and stattic, an inhibitor of signal transducer and activator of transcription 3 (STAT3), respectively, suggesting that activation of the ROS/STAT3 pathway is involved in the α-POP-induced activation of HMC3 cells. The increases in cell viability were also observed in HMC3 cells treated with other α-POP derivatives and METH.

**Conclusions:**

These results suggest that enhanced productions of ROS and IL-6 are also involved in microglial activation by drug treatment and that HMC3 cell-based system is available to evaluate accurately the microglial activation induced by abused drugs.

**Supplementary Information:**

The online version contains supplementary material available at 10.1007/s11419-024-00708-x.

## Introduction

**Fig. 1:**
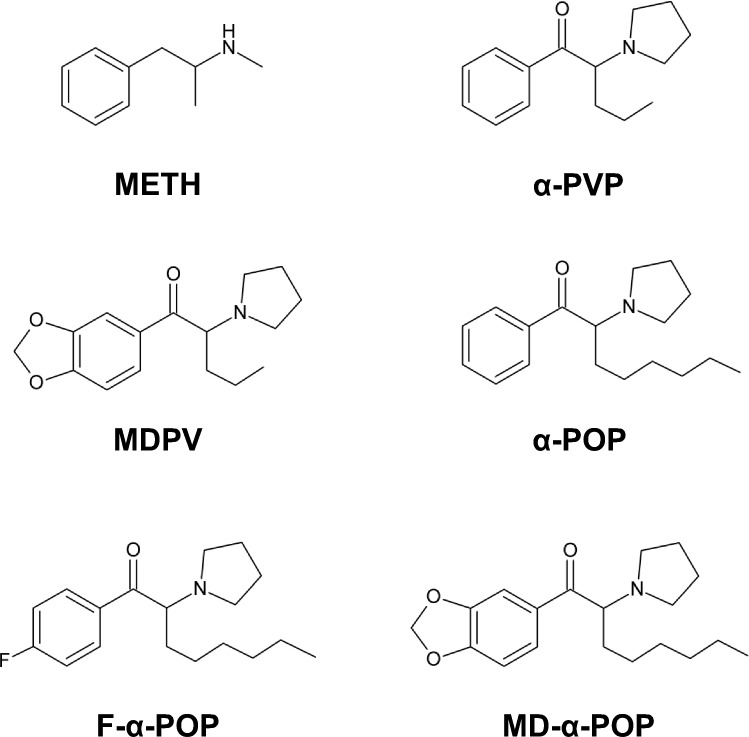
Structures of methamphetamine (METH), α-pyrrolidinovalerophenone (α-PVP), 3ʹ,4ʹ-methylenedioxypyrovalerone (MDPV), α-pyrrolidinooctanophenone (α-POP) and α-POP derivatives with 3ʹ,4ʹ-methylenedioxy ring (MD-α-POP) and 4ʹ-fluoro group (F-α-POP)

Pyrrolidinophenone derivatives (PPs), amphetamine-like designer drugs with a pyrrolidine ring, have been attracting attention in recent years owing to their worldwide distribution [1, 2]. Because of the high structural similarity to methamphetamine (METH), PPs exhibit inhibitory activity on monoamine transporters, resulting in induction of various effects similar to the stimulant drug. Since PPs in general display higher lipophilicity compared to stimulant drugs including METH, chronic administration of the derivatives raises concerns about potentially adverse effects due to an enhancement of the blood–brain barrier (BBB) penetration into the brain [3, 4]. The most prevalently abused PPs are 3ʹ,4ʹ-methylenedioxypyrrovalerone (MDPV) and α-pyrrolidinovalerophenone (α-PVP), which have been shown to induce symptoms such as tachycardia, agitation, hypertension, hallucinations, and delirium [5–7]. Additionally, chronic exposure to the derivatives has been suggested to cause disorders of the central nervous system (CNS), consequently leading to the onset and development of neurodegenerative and psychiatric diseases [2, 4, 8], as well as the indication of severe adverse effects resembling those reported for METH [9]. Literature has indicated that the CNS disorders are attributable to the BBB disruption [10, 11] and apoptosis in neuronal cells [12]. Recently, we have proposed reactive oxygen species (ROS) as a key regulator responsible for the neuronal cell damage and BBB permeability by exposure to highly lipophilic compounds such as α-pyrrolidinooctanophenone (α-POP), a highly lipophilic PP with long hydrocarbon main chain [13–16], (Fig. 1). However, little is known about the detailed mechanisms of the CNS disorders caused by exposure to PPs.

Microglia are intrinsic immune cells located within the parenchyma of the CNS [17]. In particular, the cells play central roles in the immune- and inflammation-related responses against damaged cells and xenobiotics, as well as maintenance of synaptic plasticity [17, 18]. Upon activation in response to serious adverse events such as the CNS damage, microglia are known to proliferate and undergo morphological and functional changes into M1-type cells, which have a high capacity for cytokine secretion [18]. This activation of microglia, referred to as reactive microgliosis, has been implicated in the pathogenesis of neurodegenerative and psychiatric disorders [19, 20]. Considering that administration of METH induces reactive microgliosis rather than neuronal cell death at concentrations habitually used in in vivo experiments [21–23], it is assumed that reactive microgliosis is an essential event that occurred in CNS disorders during exposure to PPs and/or pathogenesis of the above diseases. However, since there has been no in vitro system for evaluating reactive microgliosis evoked by abused drugs, accurate and easy-to-use methods to assess microglial proliferation need to be developed.

The human microglial cell line HMC3, established through SV40-dependent immortalization of human embryonic microglial cells, has been used as a microglial model since 1995 [24]. This cell line is authenticated by American Type Culture Collection (ATCC) as a microglial experimental model and is used for evaluating microglial activation [25]. A notable characteristic of HMC3 cells is their high production of a cytokine interleukin-6 (IL-6), which is known to be mainly produced by microglia and astrocytes in the brain [24, 26]. In addition, the IL-6 overproduction is observed not only in the pathogenesis of neurodegenerative diseases [27], but also in the brains of mice during the initial stages of METH administration [28] and in the serum levels of chronic METH users [29]. Although IL-6 production is thus suggested to be a key factor in microglial activation caused during exposure to the abused drug, it is unknown whether and how the IL-6 production is promoted by treating with the drug or not. In addition, in evaluation systems using HMC3 for assessing microglial activation, well-known immune activators such as lipopolysaccharide (LPS) have been commonly employed [24]. However, it remains unclear whether these systems can be applied to the evaluation of activation induced by designer drugs. In the present study, we investigated the alteration in proliferative capacity of HMC3 cells by treatment with α-POP that produces a high amount of ROS [13], to aim at developing a cytotoxicity evaluation system using reactive microgliosis. Additionally, involvement of ROS production in the HMC3 cell activation was surveyed as indicative of the cell proliferation and IL-6 production. Furthermore, we analyzed the structure–activity relationship of α-POP derivatives and METH in the activation of HMC3 cells.

## Materials and methods

### Materials

α-POP, 4ʹ-fluoro-α-POP (F-α-POP), 3ʹ,4ʹ-methylenedioxy-α-POP (MD-α-POP) and METH were purchased from Cayman Chemicals (Ann Arbor, MI, USA) and Sumitomo Pharma (Osaka, Japan). 2-(4-Iodophenyl)-3-(4-nitrophenyl)-5-(2,4-disulfophenyl)-2H-tetrazolium monosodium salt (WST-1), U0126, a mitogen-activated protein kinase (MAPK) inhibitor, and LY294002, a phosphatidylinositol-3 kinase (PI3K) inhibitor, were obtained from Fujifilm Wako Pure Chemicals (Osaka, Japan). Stattic, a signal transducer and activator of transcription 3 (STAT3) inhibitor, was from Selleck Biotech (Tokyo, Japan); *N*-acetyl-L-cysteine (NAC) was from Sigma-Aldrich (St. Louis, MO, USA); Cytotoxicity Lactate Dehydrogenase (LDH) Assay Kit-WST and NADP/NADPH Assay Kit-WST were from Dojindo (Kumamoto, Japan); NucleoSpin RNA and TB Green Premix Ex Taq were from Takara Bio (Shiga, Japan). 6-Chloromethyl-2ʹ,7ʹ-dichlorodihydrofluorescein diacetate (CM-H_2_DCFDA) and bicinchoninic acid protein (BCA) assay kit were from Thermo Fisher Scientific (Waltham, MA, USA); ECL Prime Western Blotting Detection Reagent was from GE Healthcare (Buckinghamshire, UK); Human IL-6 ELISA Kit was from Proteintech (Rosemont, IL, USA); ReverTra Ace qPCR RT Master Mix Kit was from and Toyobo (Osaka, Japan). All other chemicals were of the highest grade and could be obtained commercially.

### Cell culture

HMC3 cells and their growth medium Dulbecco's modified Eagle medium (DMEM) were purchased from ATCC (Manassas, VA, USA) and Fujifilm Wako Pure Chemicals, respectively. The cells were seeded in dishes and grown in DMEM supplemented with 10% fetal bovine serum (FBS), penicillin (100 units/mL) and streptomycin (100 µg/mL) at 37 ℃ in a humidified incubator containing 5% CO_2_. For the experiments, the cells were used at 5–15 passages and seeded at densities of 1.5 × 10^5^ cells/dish and 8 × 10^3^ cells/well into a 60-mm dish and 96-well microplate, respectively. After reaching a 70% confluence of the cells, the medium was replaced with an assay medium supplemented with antibiotics alone 2 h before treatment with agent, which was dissolved in dimethyl sulfoxide as the vehicle.

### Cell viability and cytotoxic assays

Cell viability after treatment with agents was evaluated using a formazan dye-based assay with WST-1 tetrazolium salt [30]. The median LC_50_ (lethal concentration of 50%) value was calculated from the cell viabilities after treating with incremental concentrations of the agents for 24 h. Dimethyl sulfoxide was used as a vehicle for the treatment. Cell viability was also assessed by a trypan blue dye-exclusion cell count assay. The cells were stimulated by the agents and then collected after trypsinization. After staining with trypan blue, the number of the cells excluding trypan blue was counted using a disposable hemocytometer (Funakoshi, Tokyo, Japan). Additionally, cytotoxicity was measured using an LDH assay with the Cytotoxicity LDH Assay Kit-WST.

### Measurement of ROS level and NADP^+^/NADPH ratio

Intracellular ROS level was monitored using a fluorogenic probe, CM-H_2_DCFDA. After washing with Dulbecco’s phosphate-buffered saline (DPBS), the cells treated with agents were incubated for 30 min in a fresh serum-free medium containing 20 µM fluorogenic probe. The cells were washed twice with DPBS, suspended in DPBS containing 0.1% Triton X-100, and then homogenized by passing the cell suspension through a 26-gauge needle (30 strokes). The homogenate was centrifuged at 12,000×*g* for 15 min, and the resultant supernatant was used as the sample. The intensities of fluorescence derived from DCF were measured fluorometrically. The ratio of NADP^+^ and NADPH in the sample was measured using the NADP/NADPH Assay Kit-WST according to their manufacturer’s instructions.

### Western blot analysis

Cells were washed twice with DPBS, suspended in radio-immunoprecipitation assay (RIPA) buffer (Nacarai, Kyoto, Japan) with Halt Protease and Phosphatase Inhibitor Cocktail (Thermo Fisher Scientific) and then homogenized by passing the cell suspension through a 26-gauge needle (30 strokes). The homogenate was centrifuged at 12,000×*g* for 15 min, and the resultant supernatant was used as the cell extract. The protein concentration of the cell extract was determined with the BCA protein assay reagent. The cell extracts (20 µg) were electrophoretically separated on an SDS–polyacrylamide gel (12.5%) under reducing conditions and then transferred to a Millipore PVDF membrane by electroblotting. After blocking with 0.5% bovine serum albumin or 4% Block Ace or EZ block chemi (ATTO, Tokyo, Japan), the membrane was allowed to react with primary antibodies against phosphorylated STAT3 (*p*-STAT, #9134), total STAT3 (*t*-STAT3, #4904), *p*-ERK (#9101), *t*-ERK (#4695), *p*-Akt (#9271), *t*-Akt (#4691) and β-actin (#3700, Cell Signaling Technology, Danvers, MA, USA). The immunoreactive proteins were visualized using a peroxidase-conjugated secondary antibody and the ECL Prime Western Blotting Detection Reagent. The densities of the bands were estimated using an ATTO LuminoGraph I and its attached program, CS Analyzer 4 (ATTO, Aichi, Japan).

### Level of IL-6 release in the medium

For measurement of IL-6 levels, media after treatment with agents were collected by centrifuging at 2000×*g* for 10 min and then 20 × concentrated by centrifugal ultrafiltration with Centricon YM-10 (Millipore, Bedford, MA, USA). The levels of IL-6 in the media were measured by Human IL-6 ELISA Kit (Proteintech, Rosemont, IL, USA), according to their manufacturer’s instructions.

### Quantitative reverse transcription-polymerase chain reaction (RT-qPCR) analysis

Total RNA was isolated from cells using the RNeasy Mini Kit (QIAGEN, Hilden, Germany), and single-stranded cDNA was prepared from the total RNA sample by incubation for 15 min at 37 ℃ with ReverTra Ace qPCR RT Master Mix Kit (Toyobo). The cDNA for IL-6, inducible nitric oxide synthase (iNOS), NADPH oxidase (NOX) 2, NOX4 and β-actin were amplified from the single-stranded cDNA sample by real-time PCR using a 7500 Real-Time PCR System (Thermo Fisher Scientific) with the SYBR Green II and specific primers (IL-6 forward: 5ʹ-AACTCCTTCTCCACAAGCGCC-3ʹ, IL-6 reverse: 5ʹ-TTTCACCAGGCAAGTCTCCTC-3ʹ, NOX2 forward: 5ʹ-GGTGGCATGGATGATTGCAC-3ʹ, NOX2 reverse: 5ʹ-GGCCTCCTTCAGGGTTCTTT-3ʹ, NOX4 forward: 5ʹ-AGTAGGAGACTGGACAGAACGA-3ʹ, NOX4 reverse: 5ʹ-CGCAGAGGCTGACCTCATAG-3ʹ, β-actin forward: 5ʹ-CCTGAGGCACTCTTCCAGCCTT-3ʹ, β-actin reverse: 5ʹ-TGCGGATGTCCACGTCACACTTC-3ʹ). Each expression ratio of the transcript was calculated by normalizing to the expression of β-actin as the internal standard.

### Statistical analysis

Unless otherwise noted, data are expressed as the means ± S.D. of at least three independent experiments. Statistical evaluation of the data was performed by using the unpaired Student’s *t* test and ANOVA, followed by the Tukey–Kramer test. A *p* value < 0.05 was considered statistically significant.

## Results

### Elicitation of microglial cell activation and up-regulation of IL-6 expression by α-POP treatment

When the amount of IL-6 in growth medium of human microglial cell line HMC3 cells was preliminarily measured by the ELISA kit, the IL-6 amount was much higher compared to that in the serum-free medium (Fig. S1A). Therefore, to accurately evaluate the amount and role of IL-6 in the activation of microglial cells, the serum-free medium was adopted for the assay medium in the following experiments, although the viability of HMC3 cells was reduced during culture under serum-free conditions.

**Fig. 2:**
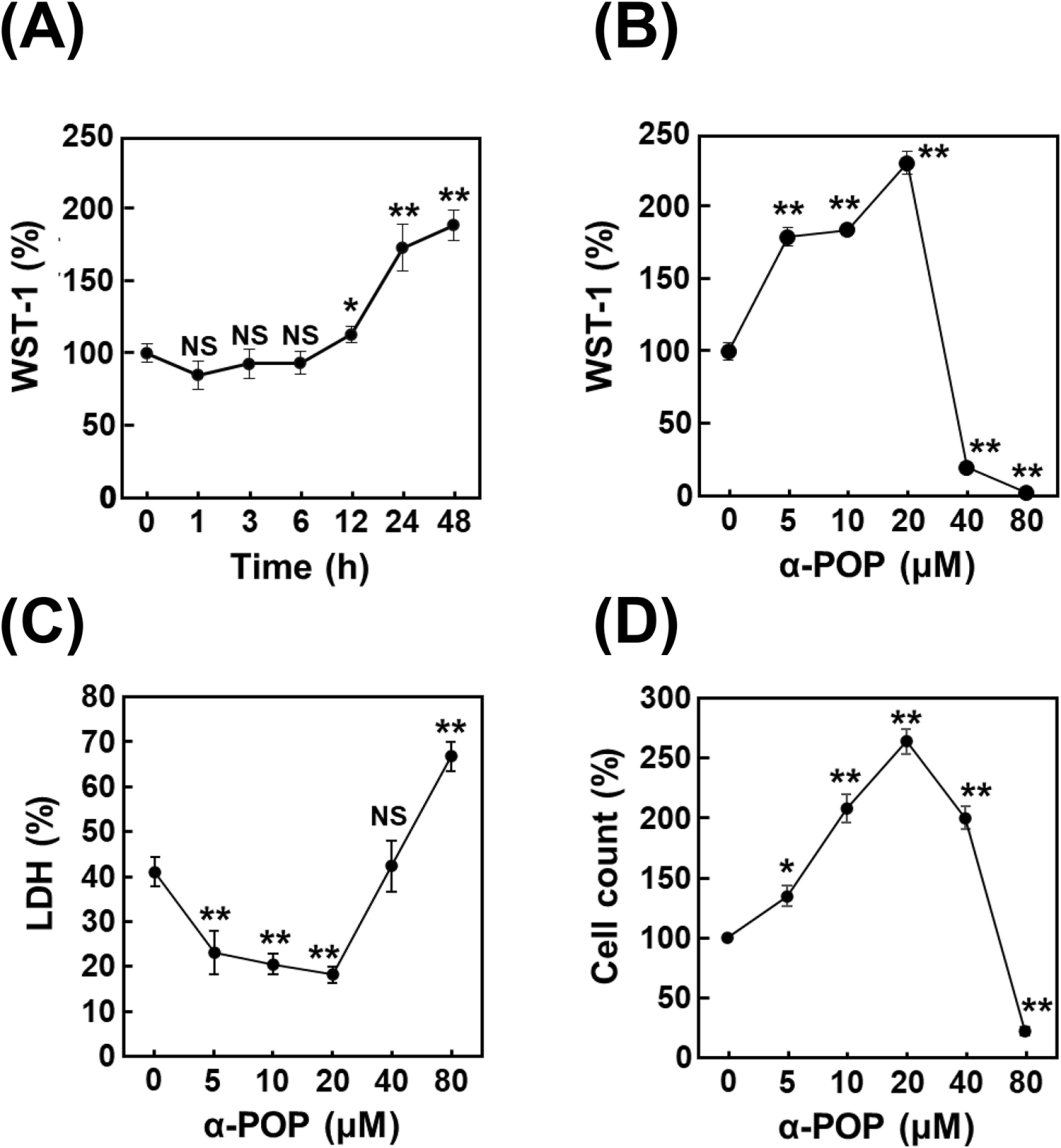
Alteration in viability of HMC3 cells by treatment with α-POP. **A** Time course experiment of HMC3 cell viability by α-POP treatment. The cells were treated for 0, 1, 3, 6, 12, 24 and 48 h with 20 µM α-POP, and the viability value was estimated by the WST-1 assay. **B** Dose–response effect of α-POP on the cell viability. The cells were treated for 24 h with the indicated concentrations of α-POP. The WST-1 values in the treated cells are expressed as the percentage of that in the control cells treated with vehicle alone (shown as 0 µM). **C** Measurement of cytotoxicity in the LDH assay. The cells were treated as described in **B** and the cytotoxicity is expressed as the percentage of total LDH activity in the confluent cells. **D** Measurement of cell number in trypan blue dye-exclusion assay. The values in the treated cells are expressed as the percentage of that in the vehicle-treated control cells. Significant difference from the control cells, *p < 0.05 and **p < 0.01. ^NS^No significant difference, *p* > 0.05

Initially, the WST-1 assay was employed to assess changes in cell viability. In our previous study, treatment with 20 µM α-POP has been shown to induce cell death of vascular endothelial cells [13]. However, treatment with 20 µM α-POP for more than 12 h significantly elevated the viability of HMC3 cells, reaching maximal levels (approximate 200%) at 24–48 h (Fig. 2A). In the dose–response experiment, the cell viability was transiently elevated after the 24-h incubation with α-POP at sublethal concentrations ranging from 5 to 20 µM, whereas it was remarkably decreased at the drug concentrations above 40 µM (Fig. 2B). These findings suggest that the WST-1 assay may be applicable for evaluating microglial activation induced by abused drugs. To clarify the mechanism underlying the alterations in the viability observed during the α-POP treatment, both LDH assay and trypan blue dye-exclusion cell count assay were conducted under the same assay conditions as the WST-1 assay. The results of both assays indicated that treatment with α-POP at the sublethal concentrations reduces the cell toxicity and enhances the proliferative capacity (Fig. 2C, D). In contrast, the cells were unable to withstand the lethal damage elicited by the drug at concentrations higher than 80 µM. These results showed that treatment with α-POP at sublethal concentrations maintains cell survival and promotes proliferation via probably microglial cell activation. The sublethal concentrations (from 5 to 20 µM) were designated as microglial cell activation concentration (MAC) in the following experiments.

**Fig. 3:**
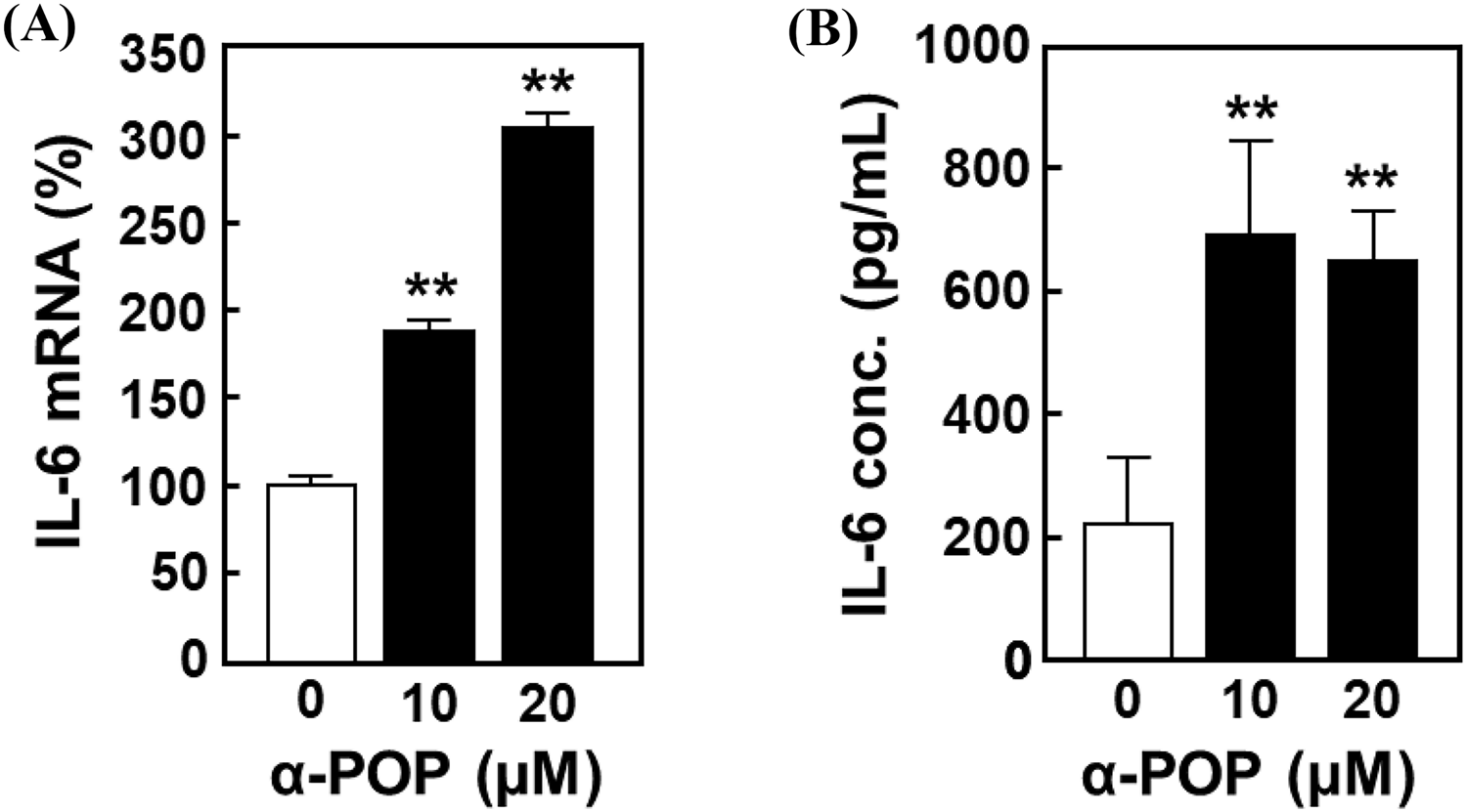
Up-regulation of expression and extracellular secretion of IL-6 by α-POP. HMC3 cells were treated for 12 h with 0, 10 or 20 µM α-POP. **A** Level of IL-6 mRNA. The expression level of IL-6 was measured by RT-qPCR analysis using its specific primers. The value in the treated cells was normalized to that of β-actin and is expressed as the percentage to that in the vehicle-treated control cells (*open bar*). **B** IL-6 concentration in the medium. The concentration of IL-6 in the medium was measured by ELISA assay and quantitated by using a linear standard curve. **Significant difference from the control cells, *p* < 0.01

To confirm whether IL-6 is involved in activation of HMC3 cells or not, alterations in expression and secretion levels of IL-6 during cell treatment with α-POP were monitored by RT-qPCR analysis and ELISA assay, respectively. The RT-qPCR analysis results showed that the α-POP treatment at low concentrations increases the expression of IL-6 mRNA in a dose-dependent manner (Fig. 3A), whereas no increase in IL-6 expression was observed with higher concentrations (80 µM) that reduced cell viability (Fig. S2). In addition, the same treatment significantly increased the amount of IL-6 released into the medium in the ELISA assay (Fig. 3B). These findings suggest that enhanced production of IL-6 is a key event that occurred during HMC3 cell activation by α-POP treatment.

### Involvement of ROS production in HMC3 cell activation by α-POP treatment

**Fig. 4:**
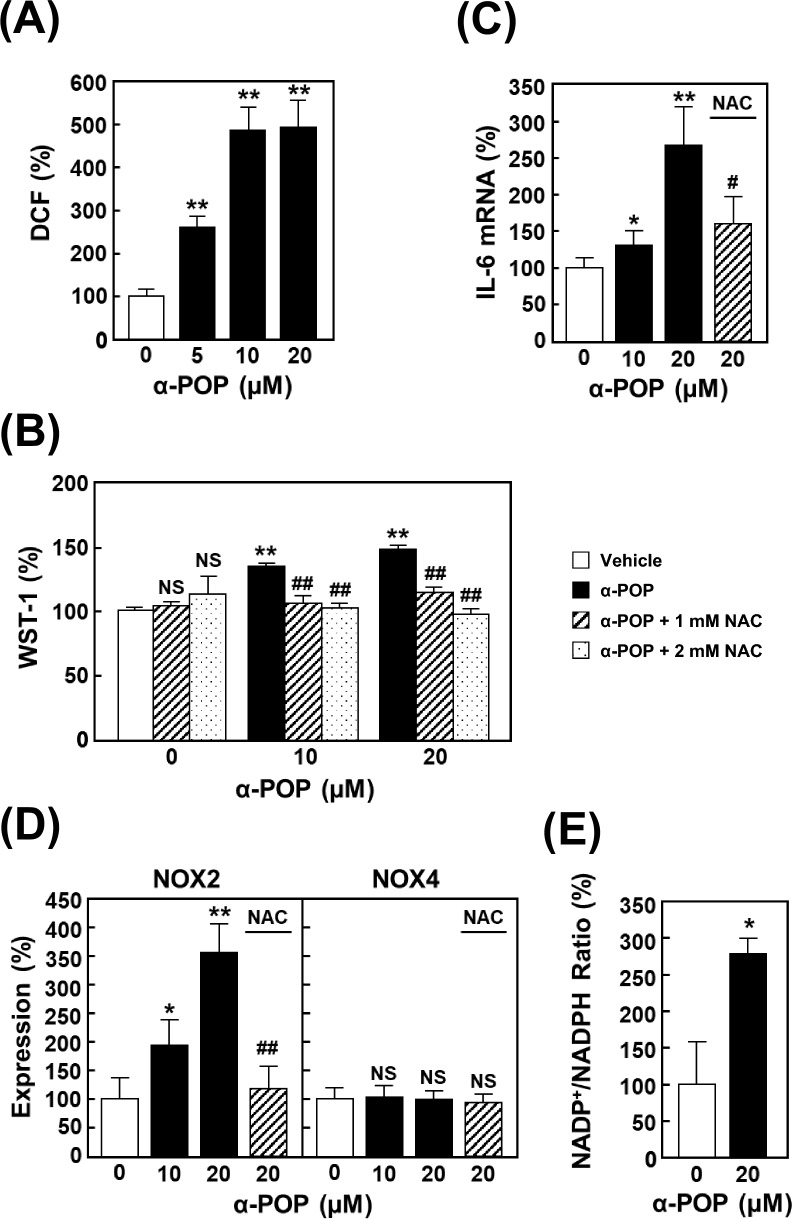
Activation of HMC3 cells due to increased ROS production by α-POP. **A** ROS production. The cells were treated for 12 h with 0, 5, 10 or 20 µM α-POP, and stained for 30 min with CM-H_2_DCFDA. The intensity of DCF-derived fluorescence is expressed as the percentage to that in the vehicle-treated control cells (*open bar*). **B** Cell viability. The cells were pretreated for 2 h with 0, 1 (*slashed bars*) or 2 mM NAC (*dotted bar*s), and then treated for 24 h with 0, 10 or 20 µM α-POP. (C and D) RT-qPCR analyses. The cells were pretreated for 2 h with 1 mM NAC (*slashed bars*), and then treated for 12 h with 0, 10 or 20 µM α-POP. The mRNA expression levels of IL-6 (**C**), NOX2 and NOX4 (**D**) in the cDNAs were measured by RT-qPCR analyses using their specific primers. The values in the treated cells were normalized to those of β-actin and are expressed as the percentages to those in the control cells (*open bars*). **E** NADP^+^/NADPH ratio. Extracts of the cells treated with 0 or 20 µM α-POP were subjected to measurement using the NADP/NADPH Assay Kit-WST. The values in the treated cells are expressed as the percentages to those in the control cells (*open bar*). Significant difference from the control cells, *p < 0.05 and **p < 0.01. Significant difference from the cells treated with α-POP alone, ^#^*p* < 0.05 and ^##^*p* < 0.01. ^NS^No significant difference, *p* > 0.05

ROS overproduction and microgliosis are linked to the pathogenesis of neurodegenerative diseases and psychiatric disorders [31]. On the other hand, moderate increases in ROS production can sometimes promote cell proliferation and differentiation through the direct phosphorylation of tyrosine kinases [32]. Considering that α-POP treatment elicits damage against various cells through mechanism dependent on ROS [13], it is possible that the ROS overproduction is responsible for inducing microgliosis by PPs. To test this possibility, the level of ROS produced in HMC3 cells by α-POP treatment was surveyed using the CM-H_2_DCFDA fluorescence probe. The ROS production was concentration-dependently increased by drug treatment (Fig. 4A). Additionally, pretreatment with the antioxidant NAC significantly restored the increase in viability values (Fig. 4B) and up-regulation of IL-6 expression (Fig. 4C) caused by α-POP, suggesting that ROS overproduction by α-POP triggers activation of HMC3 cells.

Activation of inflammation-related cells, such as microglia and macrophages, elicits enhancement of ROS production via NOX [31, 33]. As evident from the results of RT-qPCR analysis (Fig. 4D), the α-POP treatment up-regulated the expression of NOX2, but had no effect on the expression of NOX4. This up-regulation of NOX2 expression by the α-POP treatment was almost completely suppressed by pretreating with NAC. Additionally, the ratio of NADP^+^/NADPH, was significantly enhanced by the α-POP treatment, which is indicative of accelerating NADPH oxidation (Fig. 4E). These results may imply that α-POP up-regulates the NOX2 expression and consequently accelerates a positive-feedback loop of ROS production, resulting in a microgliosis.

### Involvement of the STAT3 pathway in HMC3 cell activation by α-POP treatment

**Fig. 5:**
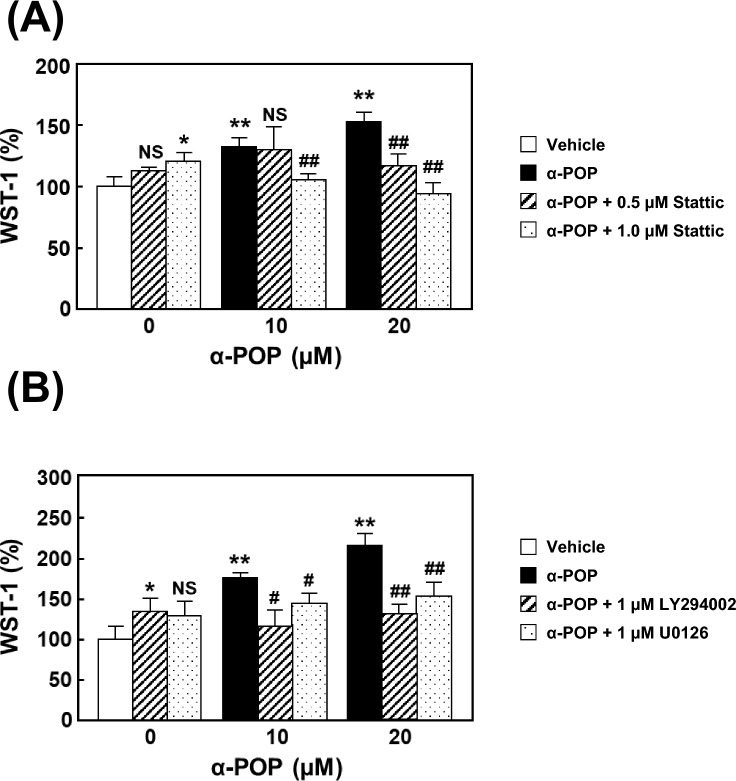
Involvement of STAT3, PI3K and MAPK in HMC3 cell activation by α-POP. **A** Effect of a STAT3 inhibitor. The cells were pretreated for 2 h with 0, 0.5 (*slashed bars*) or 1 µM Stattic (*dotted bars*), and then treated for 24 h with 0, 10 or 20 µM α-POP. **B** Effects of PI3K and MAPK inhibitors. The cells were pretreated for 2 h with the vehicle, 1 µM LY294002 (*slashed bars*) or 1 µM U0126 (*dotted bars*), prior to the 24-h treatment with 0, 10 or 20 µM α-POP. The viability value is expressed as the percentage of that in the vehicle-treated control cells (*open bar*). Significant difference from the control cells, *p < 0.05 and **p < 0.01. Significant difference from the cells treated with α-POP alone, ^#^*p* < 0.05 and ^##^*p* < 0.01. ^NS^No significant difference, *p* > 0.05

**Fig. 6:**
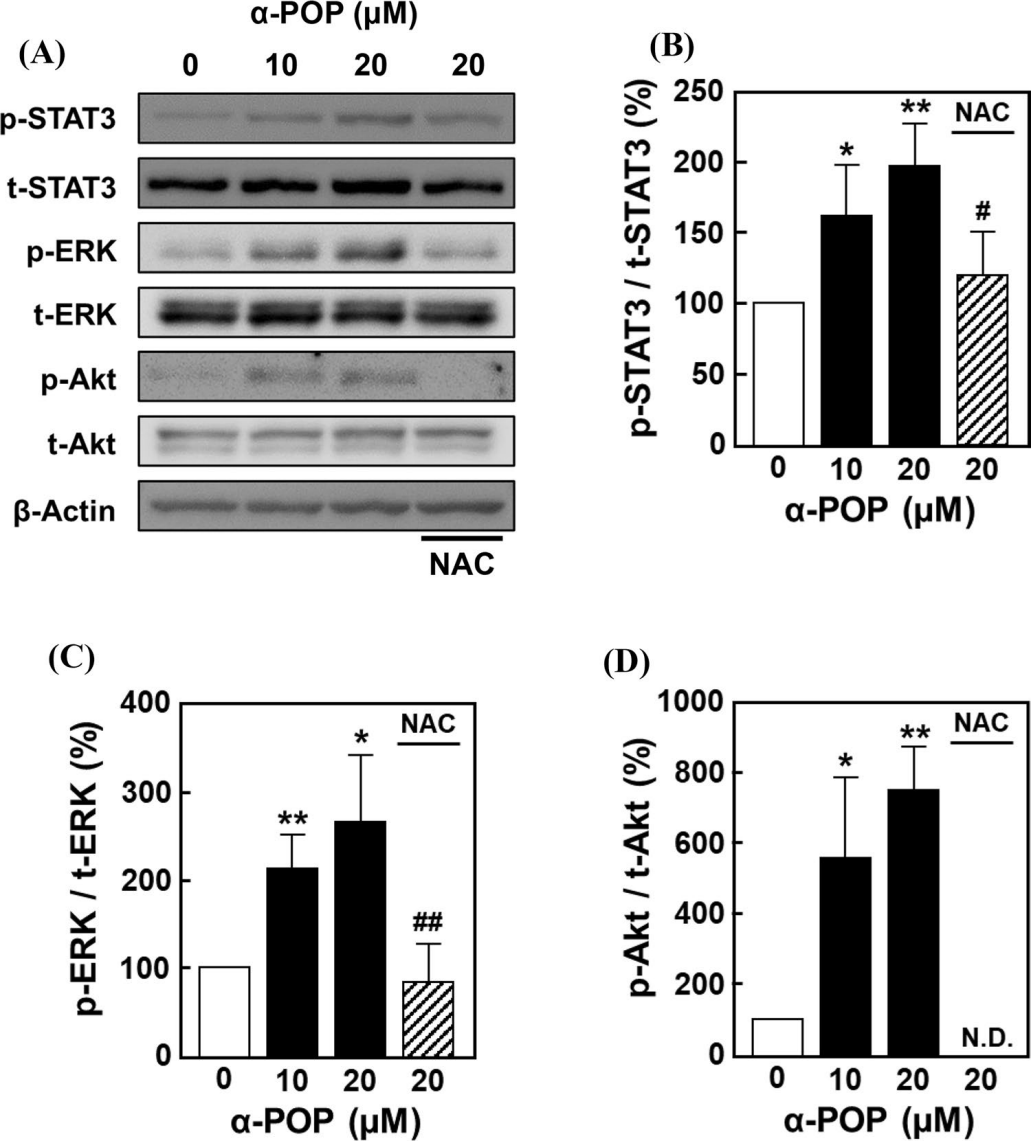
Western blot analysis of STAT3, ERK and Akt in HMC3 cells treated with α-POP. **A** Representative images. The cells were pretreated for 2 h with 1 mM NAC, and then treated for 12 h with 0, 10 or 20 µM α-POP. The cell extracts (20 µg) were applied to Western blot analysis using the antibodies against β-actin and phosphorylated (p-) and total forms (t-) of STAT3, ERK, Akt. **B**–**D** The band densities. The densities of phosphorylated forms of STAT3 (**B**), ERK (**C**) and Akt (**D**) were normalized to those of the total proteins and are expressed as the percentages of those in the vehicle-treated control cells (*open bar*). Significant difference from the control cells, *p < 0.05 and **p < 0.01. Significant difference from the cells treated with α-POP alone, ^#^*p* < 0.05 and ^##^*p* < 0.01. N.D., not detected

The extracellularly released IL-6 binds to its receptor and then activates STAT3 phosphorylation via the autocrine/paracrine pathways [34, 35]. This activation is also known to promote the positive feedback of IL-6 through the downstream signaling pathways such as MAPK and PI3K [36]. To investigate whether these pathways are involved in the activation of HMC3 cells by α-POP, HMC3 cells were pretreated with inhibitors of STAT3, MAPK, and PI3K prior to drug treatment. Pretreatment with Stattic, a STAT3 inhibitor, dose-dependently suppressed the increase in cell viability induced by α-POP (Fig. 5A). Similar recovery of the α-POP-induced effects was observed in the cells pretreated with PI3K and MAPK inhibitors, LY294002 and U0126, respectively (Fig. 5B). Additionally, in our Western blotting results, the α-POP treatment promoted the phosphorylation of STAT3, ERK, and Akt, which was significantly suppressed by pretreating with NAC (Fig. 6). These results suggest that α-POP-induced ROS overproduction promotes the HMC3 cell activation through accelerating the positive-feedback loop of the STAT3-dependent pathways (including its downstream MAPK and PI3K pathways) and IL-6 production.

### Structure–activity relationship analysis of α-POP derivatives and METH in HMC3 cell activation

**Fig. 7:**
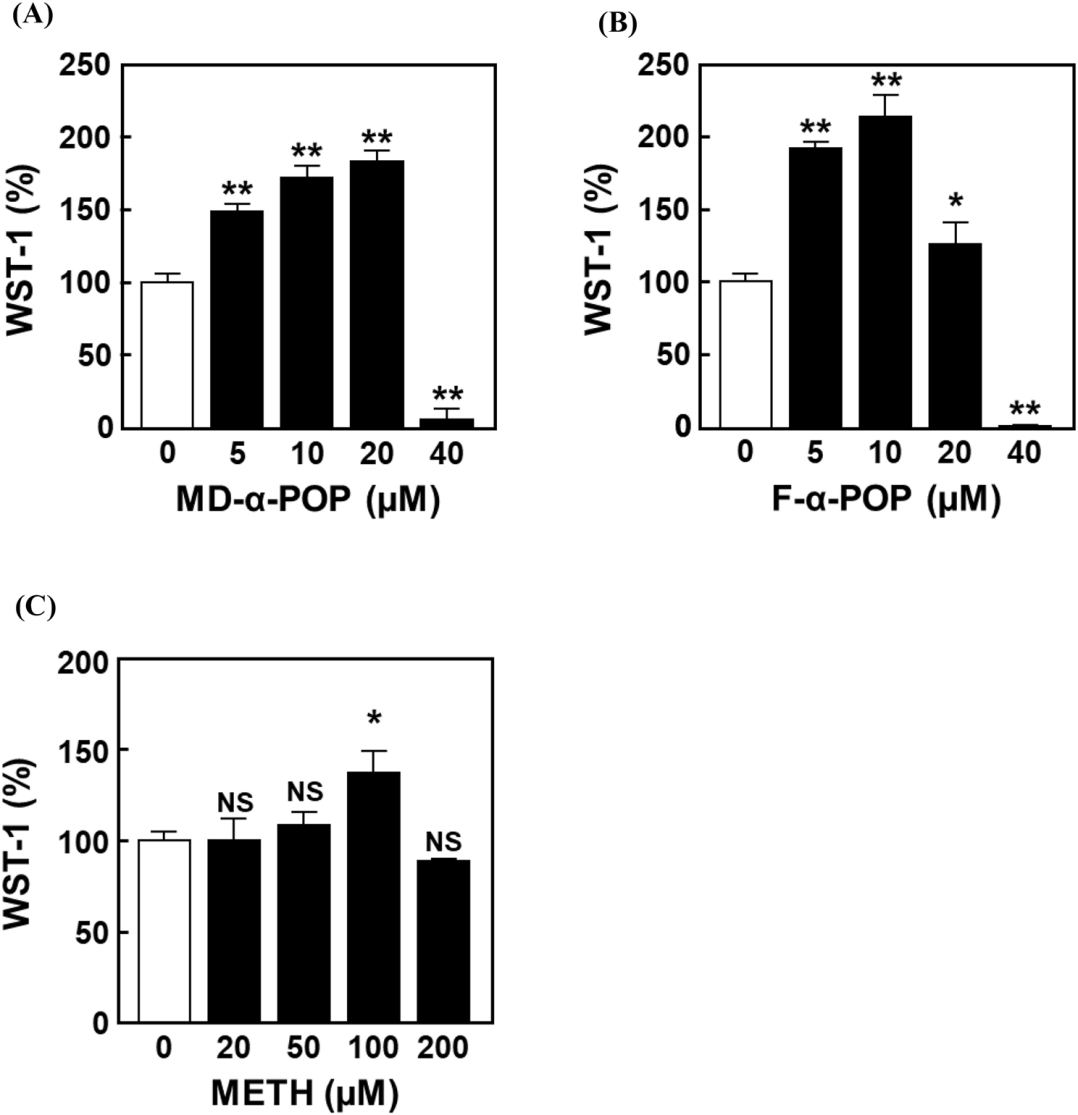
Evaluation of HMC3 cell activation by treatments with α-POP derivatives and METH. The cells were treated for 24 h with various concentrations of MD-α-POP (**A**), F-α-POP (**B**) or METH (**C**). The WST-1 values in the treated cells are expressed as the percentage of that in the vehicle-treated control cells (*open bar*). Significant difference from the control cells, *p < 0.05 and **p < 0.01. ^NS^No significant difference, *p* > 0.05

We determined whether the method for evaluating microglial activation using HMC3 cells is applicable to other PPs and METH. The structure–activity relationship analysis exhibited that the treatment with MD-α-POP and F-α-POP elicits an increase in WST-1 values at concentrations similar to α-POP (Fig. 7A, B). As shown in Table 1, the LC_50_ values of α-POP derivatives were consistent across all derivatives, suggesting that microglia are activated at concentrations of approximately one-third of the LC_50_ values of the derivatives. In contrast, the METH treatment showed an increase in WST-1 value at 100 µM (Fig. 7C), which was one-eighth of the LC_50_ value of METH. These results indicate that the ratio of MAC to LC_50_ is different in the basic structure of abused drugs. Thus, although preliminary screening is necessary to estimate the MACs, the evaluation of microglial cell activation using the HMC3 cell-based WST-1 assay is potentially useful for risk assessment of MACs of the abused drugs.

**Table 1:** LC_50_ values of METH and α-POP derivatives against HMC3 cells

	LC_50_ (µM)
METH	817 ± 38
α-POP	37.12 ± 0.36^a^
MD-α-POP	35.00 ± 0.74^a,b^
F-α-POP	32.13 ± 0.99^a,c^

^a^Significant difference from METH, *p* < 0.01

^b^Significant difference from α-POP, *p* < 0.05

^c^Significant difference from α-POP, *p* < 0.01

## Discussion

In this study, we found marked up-regulations of the proliferative capacity and IL-6 expression in HMC3 cells by treatment with PPs including α-POP at MACs and then concluded that exposure to the abused drugs facilitates reactive microgliosis. According to a case report, the postmortem blood concentration of α-POP was ~ 10 µM [37], which is in accordance with the MACs in the present study. Therefore, it is suggested that the reactive microgliosis is sufficiently caused by α-POP in the abuser. Here, we propose that the HMC3 cell-based system using WST-1 reagent is available to properly evaluate reactive microgliosis induced by PPs. Because the cell damage rate in the control group (treated with vehicle in serum-free medium) was nearly 40% in the LDH assay (Fig. 3C), this system provides the advantage of understanding the status (activation or damage) of cells exposed to abused drug in a single assay. The damage under the basal condition is likely due to apoptosis, as evidenced from the increase in caspase-3 activity shown in Fig. S1B. WST-1 and LDH assays exhibited that HMC3 cells were highly damaged during exposure to α-POP at concentrations higher than 40 µM. Our previous investigation found that incubation with a high concentration of PPs with long hydrocarbon main chain, such as α-POP, elicits neuronal cell apoptosis via a mechanism dependent on ROS overproduction [14, 38, 39]. Considering that activated microglia undergo self-regulatory apoptosis [40], it is assumed that the ROS overproduction by exposure to PPs at high concentrations (40–80 µM) activates the microglial cells and consequently elicits a self-regulatory apoptosis. The mechanisms underlying both apoptotic induction during incubation in serum-free medium and up-regulation of proliferative capacity by α-POP at MAC are subjects for future investigation.

This study demonstrated that the STAT3 pathway is involved in the regulation of IL-6 expression by α-POP in HMC3 cells. Previous literature indicated that the IL-6 expression is induced by the administration of other psychostimulants and METH [28, 29, 41], which is consistent with our findings in this study. It is well known that the NFκB pathway is located in the upstream of the STAT3/IL-6 pathway [42]. In this study, we additionally examined the mRNA expression of IL-1β and iNOS, which are direct targets of NFκB, as inflammatory markers distinct from IL-6. The results showed that the expression of IL-1β and iNOS was significantly increased by the α-POP treatment (Fig. S3), suggesting that the drug treatment elevates the transcriptional activity of NFκB. In addition, pretreatment with JSH23, an inhibitor of NFκB, suppressed α-POP-induced increase in WST-1 value (Fig. S4). On the other hand, it is reported that expression of the inflammatory markers such as IL-1β and iNOS in HMC3 cells is only at mRNA levels [24]. Therefore, it is considered that the up-regulation of IL-6 expression by α-POP in HMC3 cells is mainly attributed to the STAT3 pathway. The involvement of the NFκB pathway in other cell lines warrants further investigation to better understand its role and mechanisms.

Members in the NOX family are transmembrane proteins that play important roles in a variety of biological functions including host defense. One of the most characteristic features of the members is ROS production, which induces redox signaling and chemical modification of biomolecules, as well as host defense. Among the known seven human members, the expression levels of NOX2 and NOX4 in microglia are high and seem to be up-regulated in response to brain injury and cerebral ischemia, respectively [43]. In the present study, it is suggested that the α-POP treatment enhances the IL-6 expression through ROS overproduction mediated by NOX2 (Fig. 4). Another researcher reported that constructive expression of NOX4 is higher than that of NOX2 in HMC3 cells and suggested that NOX4 expression is mainly up-regulated by the treatment of IFN-γ [44]. This discrepancy might imply the presence of an unconventional mechanism for regulating the expression of NOX family members in response to stimulation with PPs. Here, we emphasize that the NOX2-mediated positive feedback of ROS production is responsible for ROS overproduction by exposure to PPs. The existence of the feedback loop might be accounted for by the fact that ROS overproduction is involved in the major neuronal toxicity mechanism of PPs [37, 45, 46].

To verify whether the maintenance of HMC3 cell survival can be used as an indicator of microglial activation by abused drugs, we conducted a WST-1 assay using METH. The results demonstrated that METH treatment increases WST-1 values in HMC3 cells (Fig. 7), suggesting that this experimental system may be applicable to the evaluation of other drugs including stimulant drugs, as well as PPs. Comparing the MACs with the LC_50_ values of METH and α-POP derivatives, we found that the MACs are much lower than the LC_50_ (Table 1). In this study, the MAC/LC_50_ ratio for METH was different from those for α-POP derivatives. Our previous report suggested that the cellular sensitivity to PPs is dependent on the liposolubility and retention time of the drug [16]. In addition, it was reported that IL-6 receptors [47] and toll-like receptors [48, 49] located in the plasma membrane play crucial roles in microglial activation. Therefore, the ratios might be influenced not only by the drug lipophilicity, but also by various factors involved in the interaction with extracellular receptors.

This is the first report to suggest the potential applicability of HMC3 cells for evaluating reactive microgliosis induced by abused drugs. We found that the increase in IL-6 production and the enhancement of ROS production by α-POP are involved in maintaining HMC3 cell survival. Particularly noteworthy is the existence of a close relationship between ROS and IL-6 in microglial cells, since both factors are suggested to be implicated in neurodegenerative and psychiatric disorders [50, 51]. Further elucidation of the mechanisms underlying the relationship between IL-6 and ROS in reactive microgliosis could provide insights not only into the side effects of drugs, but also into the pathogenesis and development of neurodegenerative and psychiatric disorders.

## Supplementary Information

Below is the link to the electronic supplementary material.

Supplementary file1 (PPTX 132 KB)
