# Supplementary material for: α-Pyrrolidinooctanophenone facilitates activation of human microglial cells via ROS/STAT3-dependent pathway
Source: Forensic Toxicol. 2024 Dec 9;43(1):142–54. doi: 10.1007/s11419-024-00708-x (PMC11782452; doi:10.1007/s11419-024-00708-x)
Supplement: Supplementary file 1 — Supplementary file1 (PPTX 132 KB) [file 11419_2024_708_MOESM1_ESM.pptx]

## Slide 1
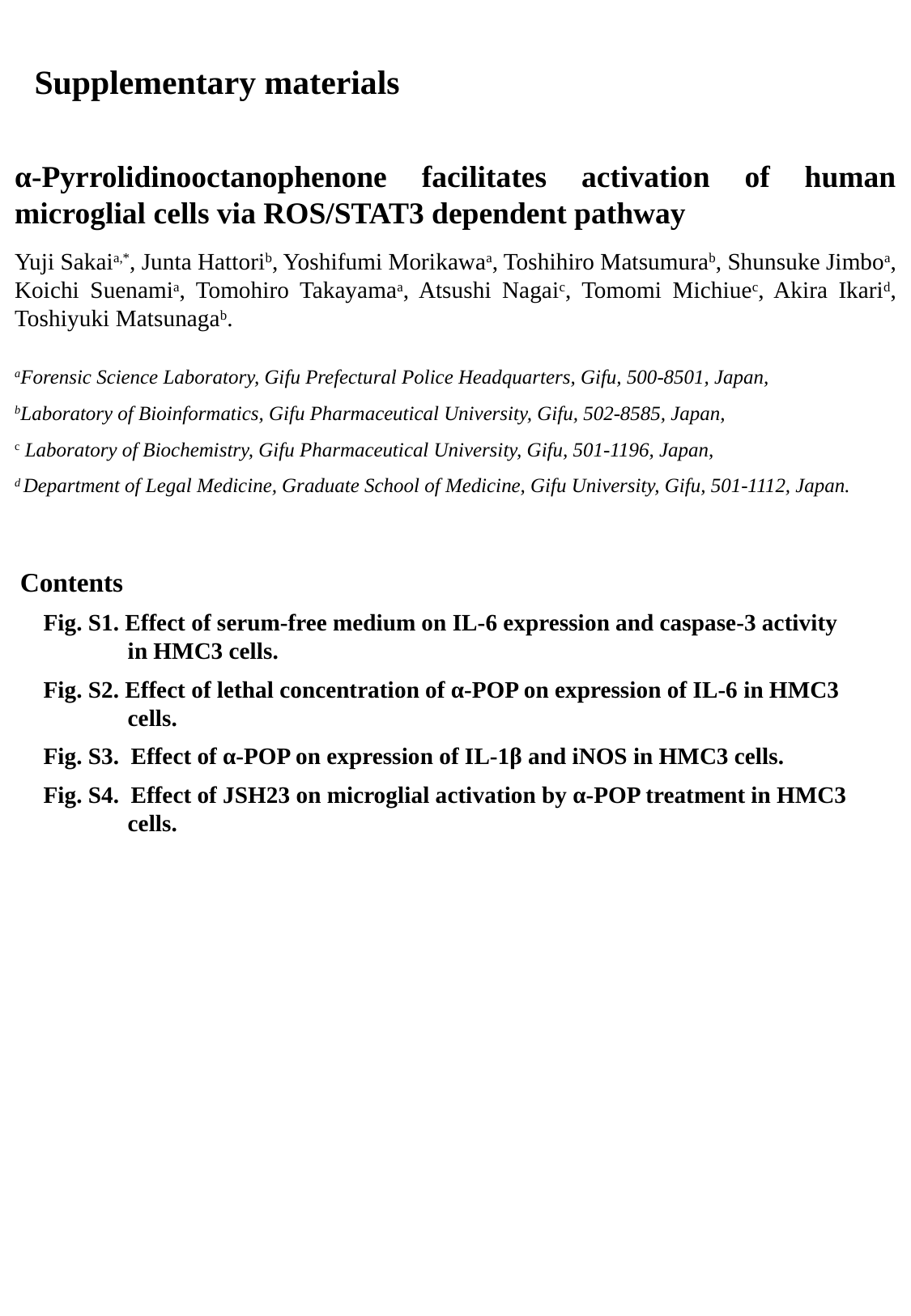

Supplementary materials
α-Pyrrolidinooctanophenone facilitates activation of human microglial cells via ROS/STAT3 dependent pathway
Yuji Sakaia,*, Junta Hattorib, Yoshifumi Morikawaa, Toshihiro Matsumurab, Shunsuke Jimboa, Koichi Suenamia, Tomohiro Takayamaa, Atsushi Nagaic, Tomomi Michiuec, Akira Ikarid, Toshiyuki Matsunagab.
aForensic Science Laboratory, Gifu Prefectural Police Headquarters, Gifu, 500-8501, Japan,
bLaboratory of Bioinformatics, Gifu Pharmaceutical University, Gifu, 502-8585, Japan,
c Laboratory of Biochemistry, Gifu Pharmaceutical University, Gifu, 501-1196, Japan,
d Department of Legal Medicine, Graduate School of Medicine, Gifu University, Gifu, 501-1112, Japan.
Contents
Fig. S1. Effect of serum-free medium on IL-6 expression and caspase-3 activity in HMC3 cells.
Fig. S2. Effect of lethal concentration of α-POP on expression of IL-6 in HMC3 cells.
Fig. S3. Effect of α-POP on expression of IL-1β and iNOS in HMC3 cells.
Fig. S4. Effect of JSH23 on microglial activation by α-POP treatment in HMC3 cells.

## Slide 2
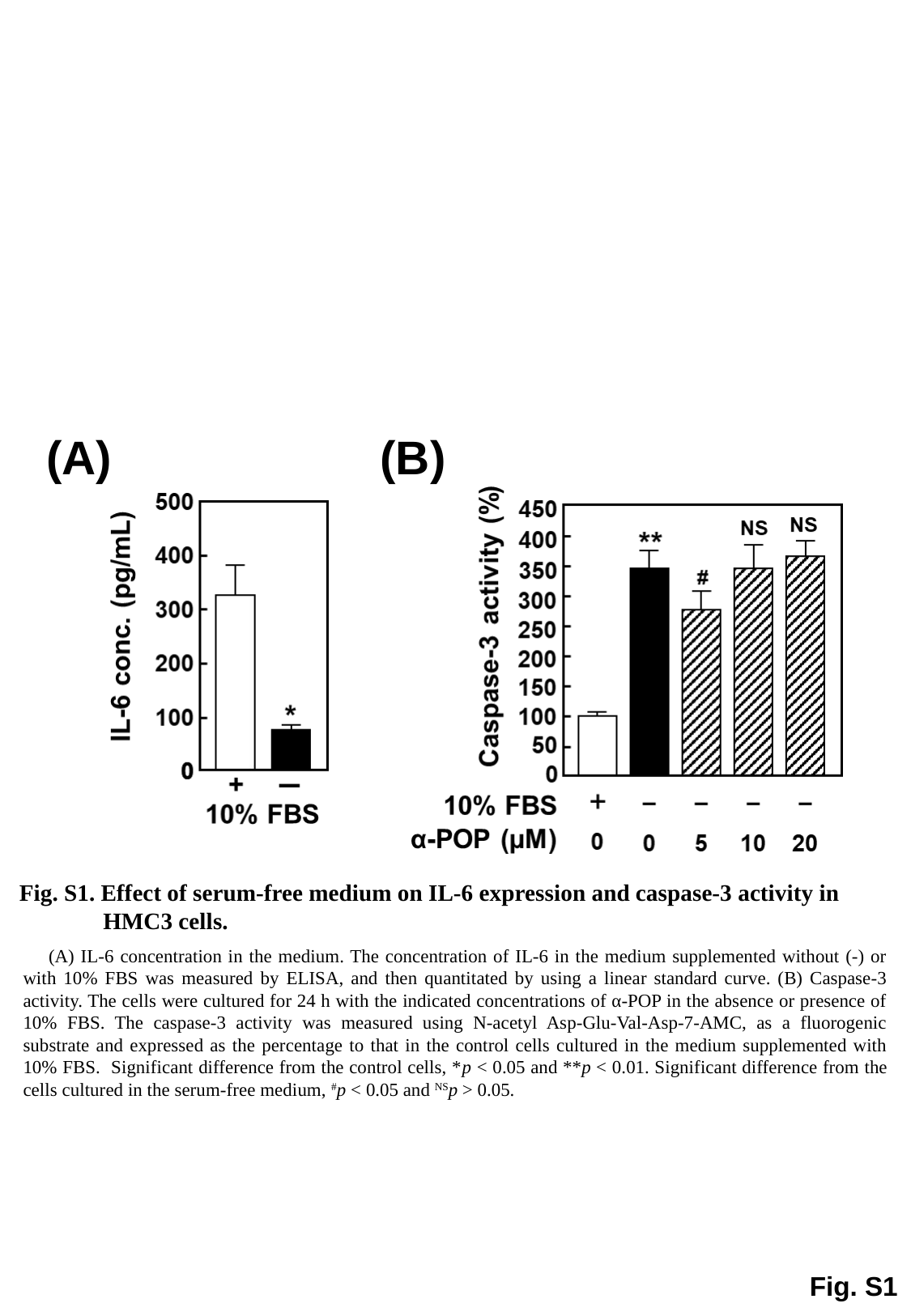

(A)
(B)
Fig. S1. Effect of serum-free medium on IL-6 expression and caspase-3 activity in HMC3 cells.
 (A) IL-6 concentration in the medium. The concentration of IL-6 in the medium supplemented without (-) or with 10% FBS was measured by ELISA, and then quantitated by using a linear standard curve. (B) Caspase-3 activity. The cells were cultured for 24 h with the indicated concentrations of α-POP in the absence or presence of 10% FBS. The caspase-3 activity was measured using N-acetyl Asp-Glu-Val-Asp-7-AMC, as a fluorogenic substrate and expressed as the percentage to that in the control cells cultured in the medium supplemented with 10% FBS. Significant difference from the control cells, *p < 0.05 and **p < 0.01. Significant difference from the cells cultured in the serum-free medium, #p < 0.05 and NSp > 0.05.
Fig. S1

## Slide 3
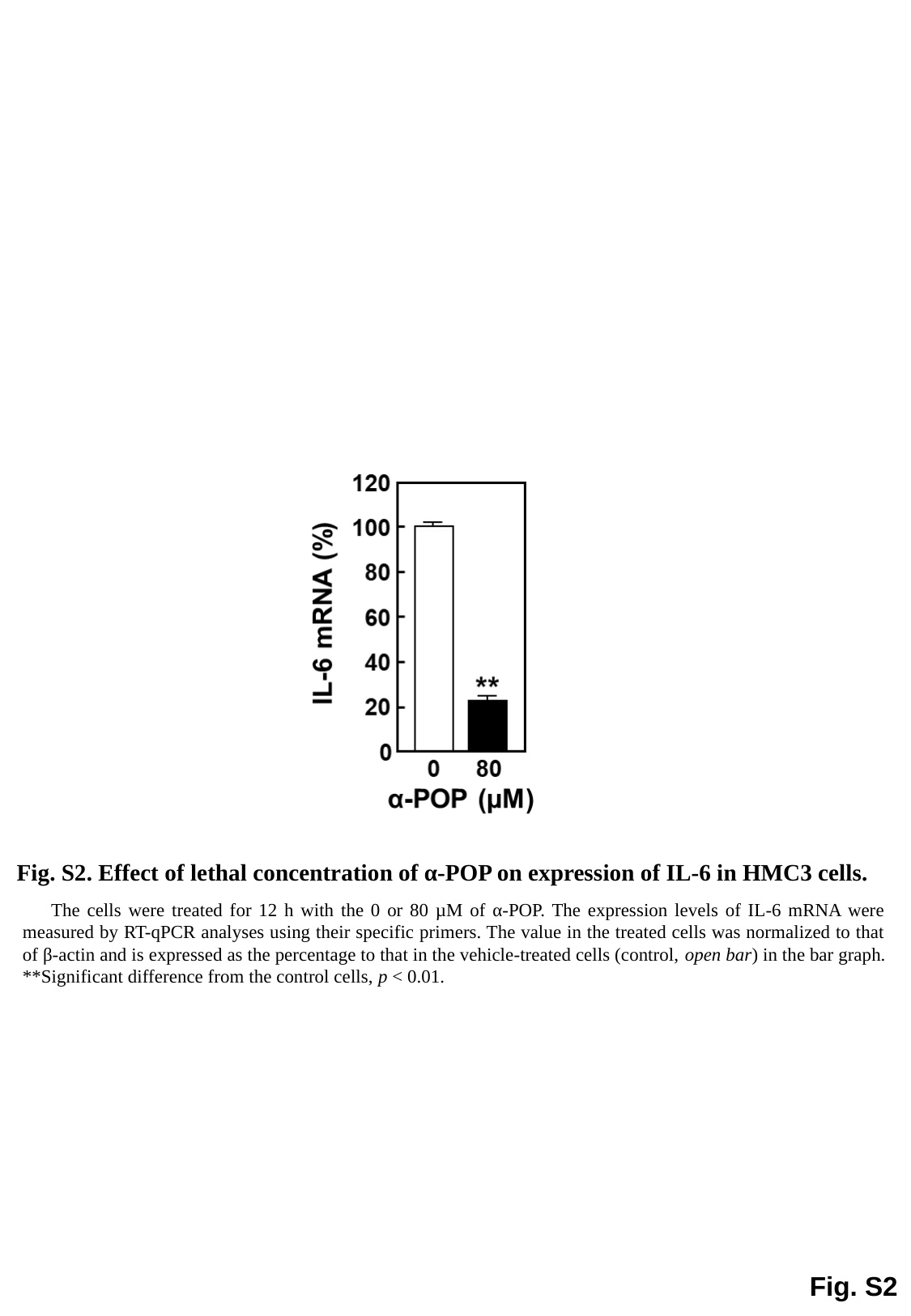

Fig. S2. Effect of lethal concentration of α-POP on expression of IL-6 in HMC3 cells.
 The cells were treated for 12 h with the 0 or 80 µM of α-POP. The expression levels of IL-6 mRNA were measured by RT-qPCR analyses using their specific primers. The value in the treated cells was normalized to that of β-actin and is expressed as the percentage to that in the vehicle-treated cells (control, open bar) in the bar graph. **Significant difference from the control cells, p < 0.01.
Fig. S2

## Slide 4
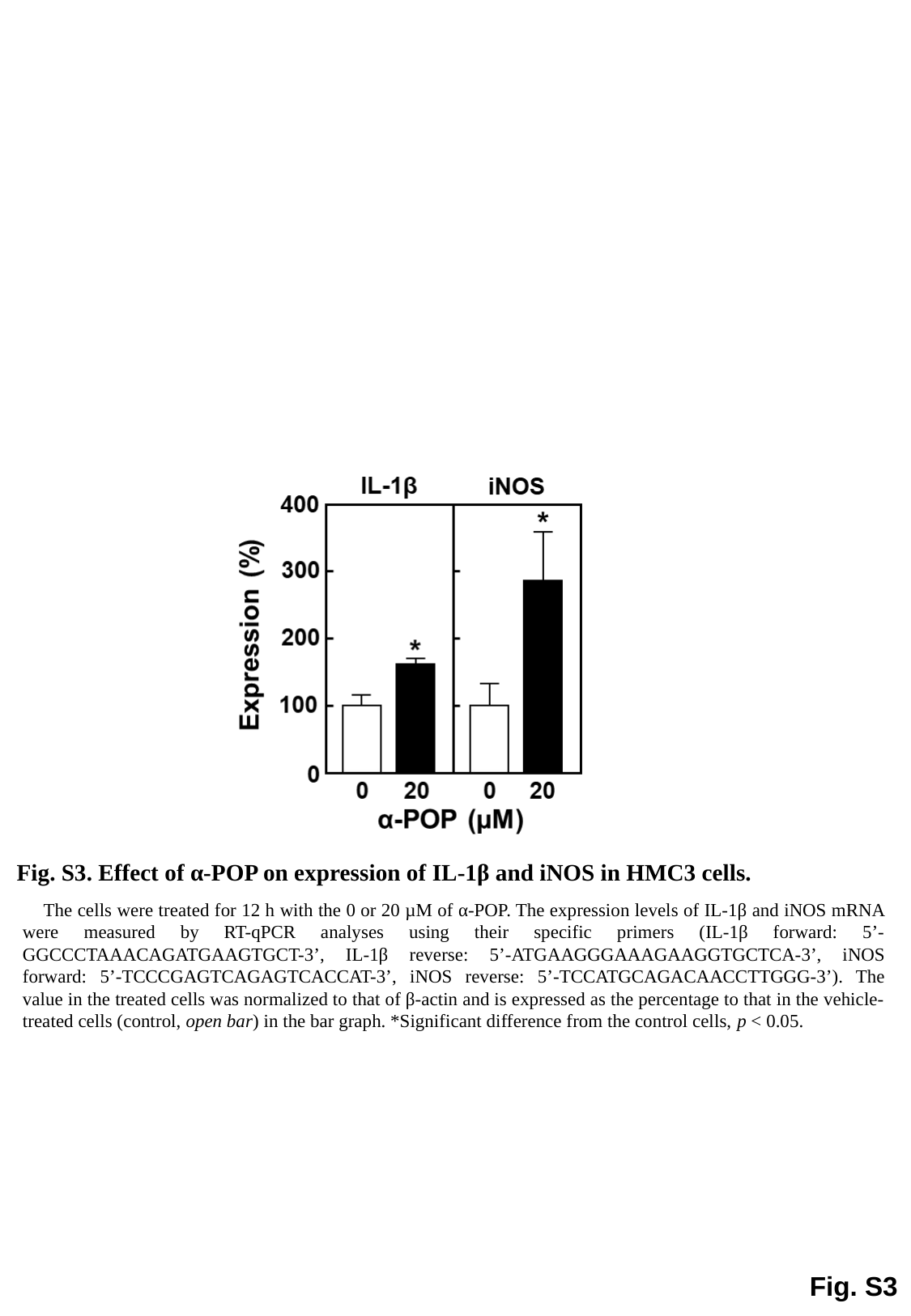

Fig. S3. Effect of α-POP on expression of IL-1β and iNOS in HMC3 cells.
 The cells were treated for 12 h with the 0 or 20 µM of α-POP. The expression levels of IL-1β and iNOS mRNA were measured by RT-qPCR analyses using their specific primers (IL-1β forward: 5’-GGCCCTAAACAGATGAAGTGCT-3’, IL-1β reverse: 5’-ATGAAGGGAAAGAAGGTGCTCA-3’, iNOS forward: 5’-TCCCGAGTCAGAGTCACCAT-3’, iNOS reverse: 5’-TCCATGCAGACAACCTTGGG-3’). The value in the treated cells was normalized to that of β-actin and is expressed as the percentage to that in the vehicle-treated cells (control, open bar) in the bar graph. *Significant difference from the control cells, p < 0.05.
Fig. S3

## Slide 5
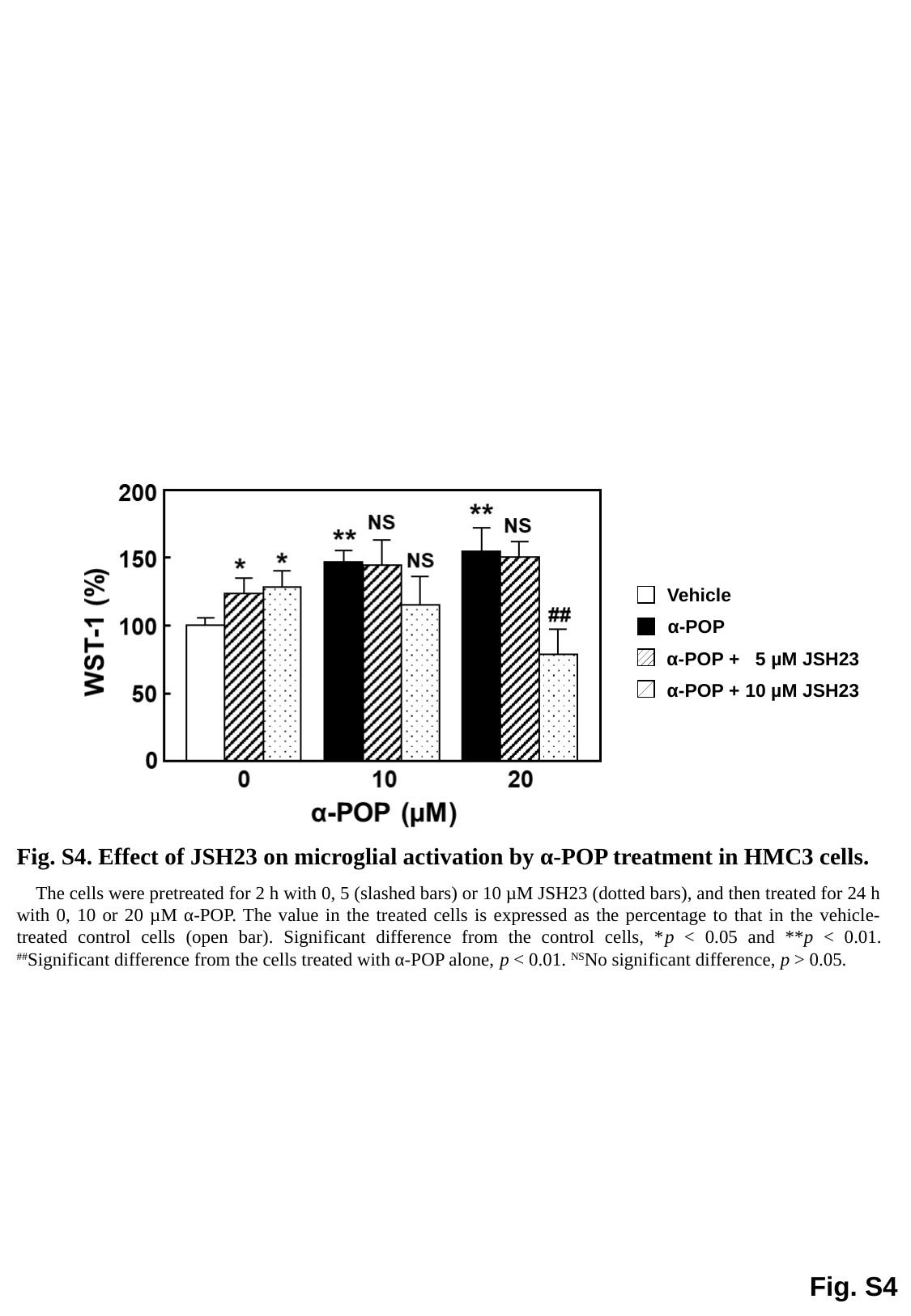

Vehicle
α-POP
α-POP + 5 µM JSH23
α-POP + 10 µM JSH23
Fig. S4. Effect of JSH23 on microglial activation by α-POP treatment in HMC3 cells.
 The cells were pretreated for 2 h with 0, 5 (slashed bars) or 10 µM JSH23 (dotted bars), and then treated for 24 h with 0, 10 or 20 µM α-POP. The value in the treated cells is expressed as the percentage to that in the vehicle-treated control cells (open bar). Significant difference from the control cells, *p < 0.05 and **p < 0.01. ##Significant difference from the cells treated with α-POP alone, p < 0.01. NSNo significant difference, p > 0.05.
Fig. S4
